# Tissue-specific roles of peroxisomes revealed by expression meta-analysis

**DOI:** 10.1186/s13062-024-00458-1

**Published:** 2024-02-16

**Authors:** Matthias Plessner, Leonie Thiele, Julia Hofhuis, Sven Thoms

**Affiliations:** 1 Department of Biochemistry and Molecular Medicine, Medical School OWL, Bielefeld University, Bielefeld, Germany; 2 Department of Child and Adolescent Health, University Medical Center, Göttingen, Germany

**Keywords:** Peroxisome, Heart, Peroxisome biogenesis, Ether lipids, Plasmalogens, Alpha-oxidation, Metabolism, Lipids, Fatty acids

## Abstract

Peroxisomes are primarily studied in the brain, kidney, and liver due to the conspicuous tissue-specific pathology of peroxisomal biogenesis disorders. In contrast, little is known about the role of peroxisomes in other tissues such as the heart. In this meta-analysis, we explore mitochondrial and peroxisomal gene expression on RNA and protein levels in the brain, heart, kidney, and liver, focusing on lipid metabolism. Further, we evaluate a potential developmental and heart region-dependent specificity of our gene set. We find marginal expression of the enzymes for peroxisomal fatty acid oxidation in cardiac tissue in comparison to the liver or cardiac mitochondrial β-oxidation. However, the expression of peroxisome biogenesis proteins in the heart is similar to other tissues despite low levels of peroxisomal fatty acid oxidation. Strikingly, peroxisomal targeting signal type 2-containing factors and plasmalogen biosynthesis appear to play a fundamental role in explaining the essential protective and supporting functions of cardiac peroxisomes.

**Supplementary Information:**

The online version contains supplementary material available at 10.1186/s13062-024-00458-1.

## Introduction

Peroxisomes are membrane-enclosed organelles present in virtually all eukaryotes [1]. Metabolic pathways in peroxisomes include reactive oxygen species (ROS) and D-amino acid (D-AA) catabolism, very long and branched-chain fatty acid (FA) oxidation, biosynthesis of bile acids (BAs), poly-unsaturated FAs (PUFAs), and plasmalogens [1]. Contact sites with other organelles, e.g., mitochondria, the endoplasmic reticulum (ER), lysosomes, and lipid droplets, enable metabolite flux [2]. Precise metabolic functions, abundance, and intracellular location of peroxisomes vary depending on tissue and species [1]. Peroxisomal biogenesis relies on a set of proteins termed peroxins (PEX) governing individual aspects of peroxisome de novo formation, fission, and the import of matrix and membrane proteins [3]. Several fission factors are shared between mitochondria and peroxisomes [4]. Peroxisome homeostasis is further maintained by pexophagy, a dedicated autophagy pathway [5]. In humans, peroxisomal dysfunction relates to more than 20 congenital diseases caused by single enzyme defects or peroxisome biogenesis disorders (PBDs) [6]. PBDs comprise the Zellweger syndrome spectrum (ZSS) of severe inheritable neuro-metabolic disorders and rhizomelic chondrodysplasia punctata (RCDP).

The majority of research on human peroxisomes centers on cerebral, hepatic, and renal tissues, that are known to be affected in PBDs. More recent studies focus on peroxisomes in other organs [7–10] including the human heart [11, 12]. Cardiomyopathies and heart failure were observed in patients with RCDP and Refsum disease (RD), both presenting with milder neurological symptoms than ZSS [13, 14]. In addition to congenital disorders with primary peroxisomal defects, peroxisomes are involved in the pathophysiology of various, more common conditions [15–17], e.g. inflammation, ischemic reperfusion, tumorigenesis, and Diabetes mellitus [18]. The tissue and disease specificity of peroxisomal metabolism is, however, largely enigmatic.

Peroxisomal biosynthesis pathways include plasmalogens, PUFAs [19], and BAs [20]. Plasmalogens are distinct phospholipids with a vinyl ether-linked fatty alcohol [21–23], and enriched in the myelin sheath, immune and cardiac cells [24]. In contrast to the kidney and liver, which provide metabolite supply, biotransformation, and excretion, ATP production in the brain and heart maintains action potentials and cardiomyocyte contraction. Cerebral production of ATP is mainly fueled by carbohydrates [25], in contrast to the metabolically omnivorous cardiomyocyte. Adult myocardial tissue favors FAs [26], highlighting the crucial role of FA oxidation in physiological heart function. Inversely, a metabolic shift towards glycolysis correlates with heart failure [27]. Most, medium- and long-chain FAs are metabolized by mitochondrial β-oxidation. Exotic FAs can only be imported in peroxisomes [28, 29]. Very long-chain FAs (VLCFAs) are metabolized by peroxisomal β-oxidation [2], and branched-chain FAs (BCFAs) by α-oxidation in peroxisomes [30]. Despite the unique features of peroxisomal FA metabolism, we know little about their contribution to cardiac health.

In this study, we evaluate the tissue-specific impact of peroxisomal metabolism with a focus on the heart. For this, we assessed the expression level of genes involved in peroxisomal biogenesis, mitochondrial and peroxisomal fission, pexophagy, metabolite flux, ROS and D-AA metabolism, mitochondrial and peroxisomal β-oxidation, peroxisomal α-oxidation and biosynthesis of BAs, PUFAs, and plasmalogens. We focused the meta-analysis on humans and mice. Aligning with the cerebrohepatorenal symptoms of ZSS and other PBDs, we analyze the brain, liver, and kidney for comparison with cardiac tissue.

## Methods

### Identification and selection of pathway-related genes

**Fig. 1:**
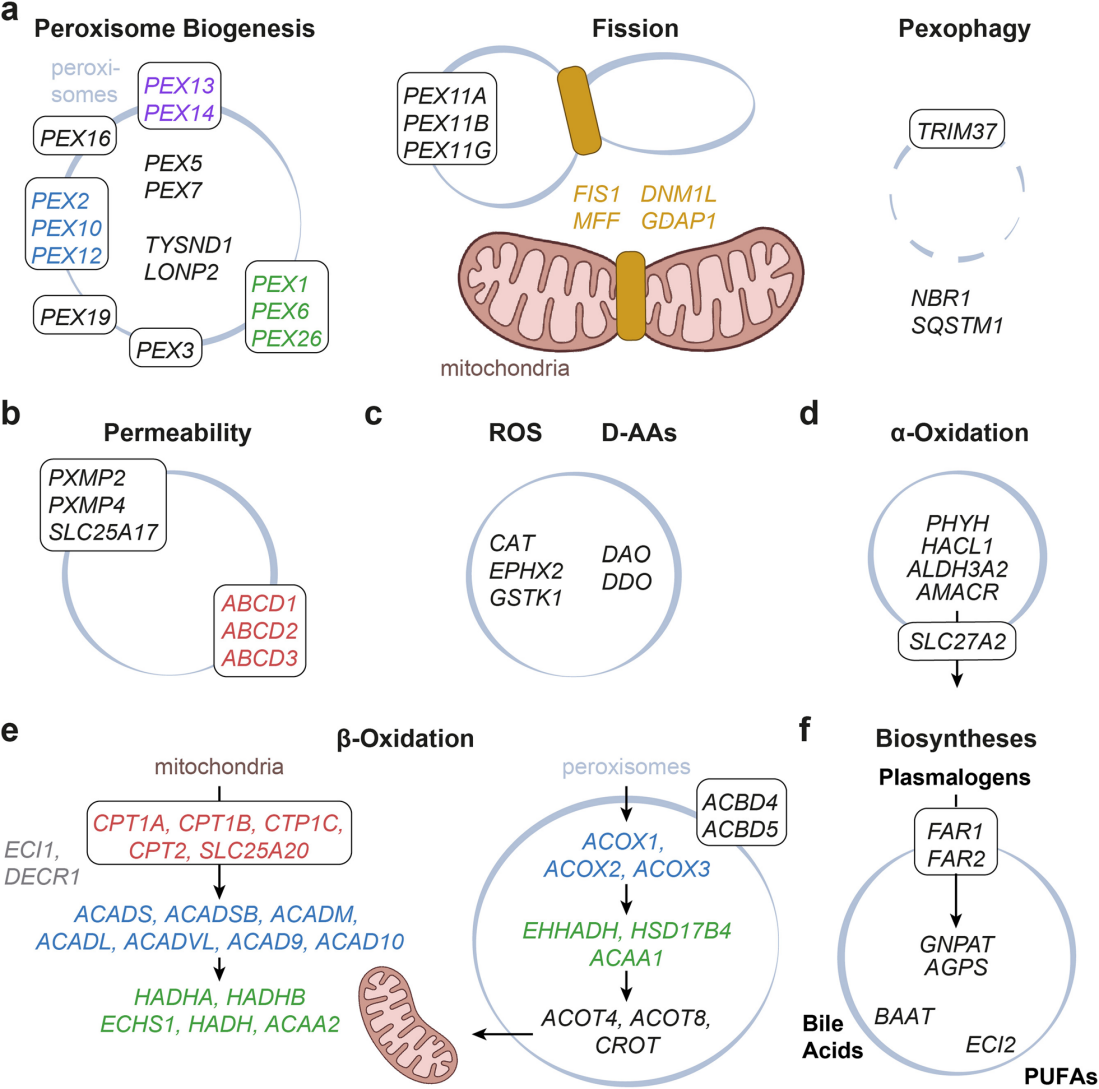
Pathway-related genes in peroxisomes and mitochondria. For simplicity, only human gene symbols are shown within the figure. **a** Mammalian peroxisome biogenesis factors include matrix protein receptors PEX5 and PEX7, membrane biogenesis factors PEX3, PEX16, and PEX19; docking and import complex components PEX13 and PEX14; E3 ubiquitin ligase complex PEX2, PEX10, and PEX12; the AAA complex PEX1 and PEX6 with tail-anchored PEX26. TYSND1 and LONP2 process imported matrix proteins. The *PEX11* family is involved in the elongation of peroxisomes; the fission of peroxisomes and mitochondria requires *FIS1*, *MFF, DNM1L,* and *GDAP1*. E3 ubiquitin ligase TRIM37 and autophagy receptors NBR1 and SQSTM1 mediate pexophagy. **b** Metabolite flux in peroxisomes relies on pore formation by PXMP2, PXMP4, and SLC25A17. ABCD1, ABCD2, and ABCD3 import FAs with different substrate selectivity. **c** *CAT*, *EPHX2*, and *GSTK1* facilitate ROS metabolism, and D-AAs are oxidized by DAO and DDO. **d** BCFAs are metabolized by PHYH, HACL1, and ALDH3A2. SLC27A2 activates and transports BCFAs. AMACR converts the (2*R*)-enantiomer. **e** Mitochondrial β-oxidation requires FA import by the carnitine shuttle CPT1A, CPT1B, CPT1C, CPT2, and SLC25A20 (red), see **b** for peroxisomal import factors. Mitochondrial import is followed by dehydrogenation (blue) by ACADS, ACADSB, ACADM, ACADL, ACADVL, ACAD9, or ACAD10; the peroxisomal analogs are ACOX1, ACOX2, and ACOX3. Mitochondrial ECI1 and DECR1 break down unsaturated FAs by isomerization and oxidation of double bonds. Hydration, oxidation, and thiolytic cleavage are carried out by multifunctional or individual enzymes (green): HADHA, HADHB, ECHS1, HADH, ACAA2 in mitochondria, and EHHADH, HSDB174, ACAA1 in peroxisomes. ACOT4 and ACOT8 catalyze the hydrolysis of acyl-CoAs into free FAs and CoA. CROT catalyzes the export of peroxisomal FAs. ACBD4 and ACBD5 tether peroxisomes to the ER. **f** Plasmalogen synthesis involves FAR1- or FAR2-mediated reduction of acyl-CoAs to primary fatty alcohols and synthesis of an ether-linked intermediate by GNPAT and AGPS. The peroxisomal enzymes, BAAT and ECI2 are essential for BA and PUFA synthesis, respectively

For a complete list of peroxisomal proteins, we summarized individual sources from a collection of peroxisomal proteome studies [31], the Compartments Knowledge Channel [32], and the UniProtKB database [33] (Additional file 2: Table S1). We identified pathway-related genes using a literature review [1, 15, 34]. After cross-reference, we selected 58 peroxisomal genes for expression meta-analysis (Fig. 1, Additional file 2: Table S1). In addition, 19 genes were selected covering mitochondrial β-oxidation (Fig. 1d, Additional file 2: Table S1) by literature review only [35].

### Data collection

We collected transcriptome and proteome expression data as averages weighted by sample size. Transcriptome data were gathered across cardiac, cerebral, hepatic, and renal tissue in postnatal humans and mice from two databases, The Human Protein Atlas (HPA) [36] and Evo-devo [37]. HPA was queried by API (application programming interface), and Evo-devo was accessed in a TSV (tab-separated value) format. At least 35 individual values were summarized per gene and tissue as RPKM (reads per kilobase per million) values. Proteome data from heart, brain, liver, and renal tissue in humans and mice were collected from ProteomicsDB as iBAQ scores (intensity-based absolute quantification) [38]. We included HPA and ProteomicsDB in our study due to comprehensive collections of human and murine gene expression data. Evo-devo provides additionally well-curated developmental transcriptome data. Single-cell mRNA expression data for cardiac tissue were obtained from Heart Cell Atlas v2 [39] in the H5AD format. The Heart Cell Atlas v2 represents an extensive resource for single-cell, cardiac transcriptome data and was therefore used to specifically analyze spatial transcriptomics in the heart.

### Clustering of mRNA expression during human tissue development

We generated a Python script training a SOM (self-organizing map) model to cluster mitochondrial and peroxisomal genes in the human Evo-Devo data set using the MiniSOM Python library (Vettigli G., https://github.com/JustGlowing/minisom, accessed on December 2nd 2023). The normalized mRNA expression averages of developmental clusters are visualized as DTW (dynamic time warping) barycenters of allocated genes (Fig. 6a). Tissue distribution is defined as the AUC (area under the curve) per tissue divided by the total AUC per cluster. We calculated normalized correlation coefficients as the mean-adjusted sum product of paired tissue distributions in developmental clusters and pathways (Fig. 6b).

### Data visualization and statistical analysis

**Fig. 2:**
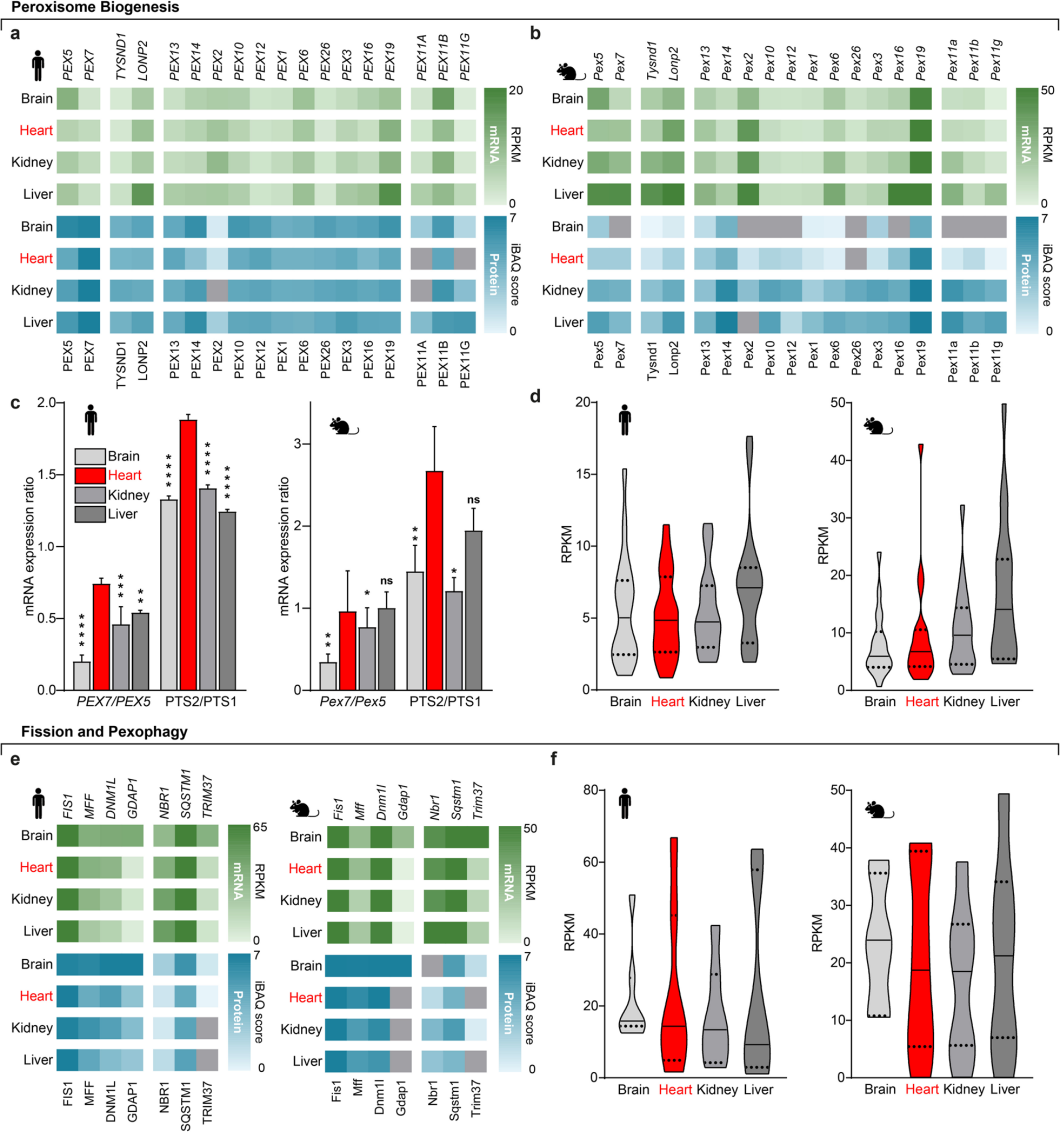
Peroxisome biogenesis, fission, and pexophagy factors are uniformly expressed across different tissues. **a, b** Heatmaps showing transcriptome (green) and proteome data (blue) of **a**) humans and **b** mice for indicated genes and proteins. Gray indicates missing values. **c** Calculated ratios for *PEX7*/*PEX5* and PTS2/PTS1-cargos of human and murine transcriptome data with indicated significant differences relative to cardiac tissue. Error bars represent SEM. ns, not significant; *p < 0.05; **p < 0.01; ***p < 0.001; ****p < 0.0001. **d** Distributions of human and murine transcriptome data for all peroxins. **e** Human and murine transcriptome (green) and proteome (blue) data, and **f** summarized distributions of human and murine transcriptome data for pexophagy, the mitochondrial, and peroxisomal fission complex

Arithmetic means of species- and tissue-specific gene expression data were calculated and visualized in heatmaps, color-coded green for transcriptome, and blue for proteome data. Color intensities reflect expression levels within each figure. The color intensities of data outliers determined by Grubb’s tests [40] are clipped at indicated maxima. Missing values are colored gray. We visualized selected species- and tissue-specific transcriptome data in violin plots to emphasize data distribution. Significant differences in Fig. 2c were determined by a two-way ANOVA adjusted for multiple comparisons using the Holm-Šidák method [41]. Differential gene expression in heart regions was determined by unpaired multiple *t*-tests using the FDR (false-discovery rate) approach [42] with α = 0.01 (Fig. 6c–g). Statistical analysis and data visualization were performed using Prism 10 (GraphPad) and Python. Figure 1 uses graphical elements from BioRender.com. Other graphical elements were created using Illustrator (Adobe).

## Results

### Identifying and selecting pathway-related genes

We started by creating sets of genes that were specific for peroxisomal metabolic or—in the broadest sense of the term—biogenetic pathways. We collected pathway-related genes using literature reviews including proteome studies and databases [1, 31, 33, 34]. A summary of peroxisome proteomics revealed 225 individual and 23 overlapping proteins indicating core and specific peroxisomal proteomes (Additional file 2: Table S1). In total, we selected 77 genes, which were allocated to respective pathways (Fig. 1, Additional file 2: Table S1). We schematically divided 47 peroxisomal genes into biogenesis, pexophagy, and fission (Fig. 1a), metabolite flux (Fig. 1b), ROS and D-AA metabolism (Fig. 1c), α-oxidation (Fig. 1d), biosynthesis of plasmalogens, PUFAs, and BAs (Fig. 1f). For β-oxidation, we included 19 mitochondrial and 11 peroxisomal genes that would allow a direct comparison of these pathways (Fig. 1e). Other genes were omitted due to indistinct pathway allocation or multiple cellular locations, e.g., *SCP2, CRAT*, *ACOT1*, *ACOT2*, *ACOT5*, *ACSL1*, *ACSL4*, *PRDX5*, *MLYCD*, *HMGCL*, *IDH1, DHRS7B,* and *SEHRL2*.

### Peroxisome biogenesis, fission, pexophagy

We analyzed 16 *PEX* genes that facilitate different aspects of peroxisome biogenesis (Fig. 1a). The peroxisomal protein PEX5 is the essential import receptor of matrix proteins and has two isoforms: PEX5S for PTS1 (peroxisomal targeting signal 1)-containing proteins, and PEX5L, the co-receptor for the PEX7-dependent import of PTS2-proteins, e.g., ACAA1, AGPS, and PHYH [43]. Both isoforms are summarized in our analysis.

Cerebral *PEX5* but not *PEX7* expression in humans is markedly elevated (Fig. 2a), indicating a preference for PTS1-proteins in the brain. This surprising, apparently tissue-specific, expression of a ubiquitous peroxisome import receptor prompted us to calculate the ratios of *PEX7*/*PEX5* and *PTS2/PTS1* mRNA transcripts. We find cerebral *PEX5* enrichment over *PEX7* (Fig. 2c) and, even more surprising, a cardiac preference for *PEX7* and associated PTS2-cargoes in human and mouse (Fig. 2c).

*PEX19* shows the highest expression while mRNA and protein expression levels of most other *PEX* genes are evenly distributed indicating a similar peroxisome biogenesis capacity across all tissues (Fig. 2a, b, d). TYSND1 and LONP2*,* involved in the post-processing of peroxisomal matrix proteins [44, 45], are elevated in the liver (Fig. 2a, b). Out of the three paralogous *PEX11* genes present in most vertebrates [46, 47], *PEX11B* shows high expression in humans (Fig. 2a), but not in mice (Fig. 2b) in line with rodent-specific, peroxisome proliferator-activated receptor-dependent regulation [48, 49]. Genes mediating mitochondrial and peroxisomal fission, and pexophagy show no tissue specificity [50] (Figs. 1a and 2e,f), except for the neuronal fission factor *GDAP1* [4], and cerebral enrichment of the E3 ubiquitin ligase *TRIM37* (Fig. 2e, f), which corresponds to the primary neurological phenotype in mulibrey nanism [51].

### Factors involved in metabolite flux, ROS, and D-AAs

**Fig. 3:**
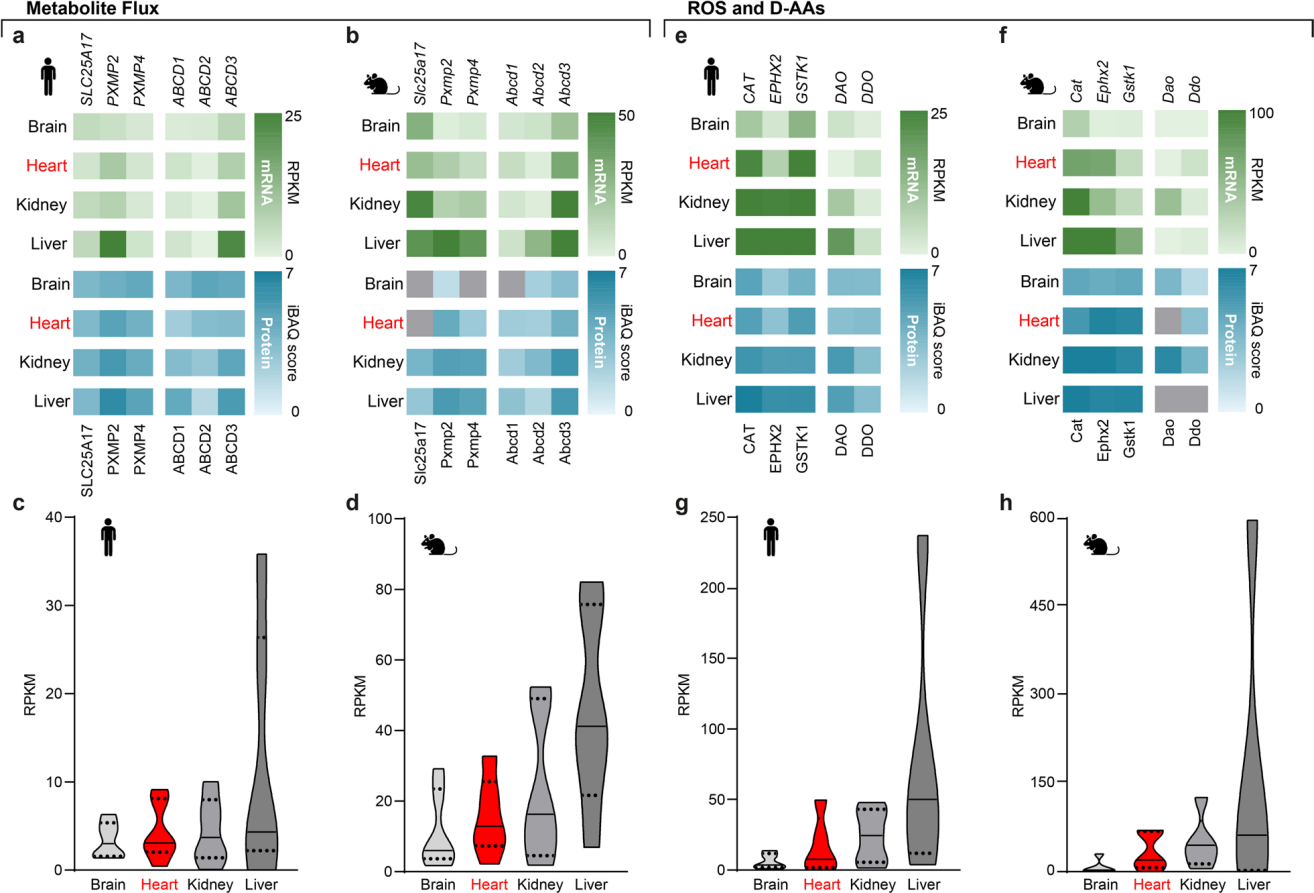
Factors for peroxisomal metabolite flux, ROS, and D-AA metabolism are equally distributed except for low cerebral ROS factors and higher expression in the liver and murine kidney. **a–d** Transcriptome (green) and proteome (blue) data in **a** humans and **b** mice together with respective summarized distributions in **c** humans and **d** mice for peroxisomal metabolite flux. **e–h** Heatmaps visualizing transcriptome (green) and proteome (blue) in **e** humans and **f** mice with summarized distributions of transcriptome data in **g** humans and **h** mice of peroxisomal factors involved in ROS and D-AA metabolism

**Fig. 4:**
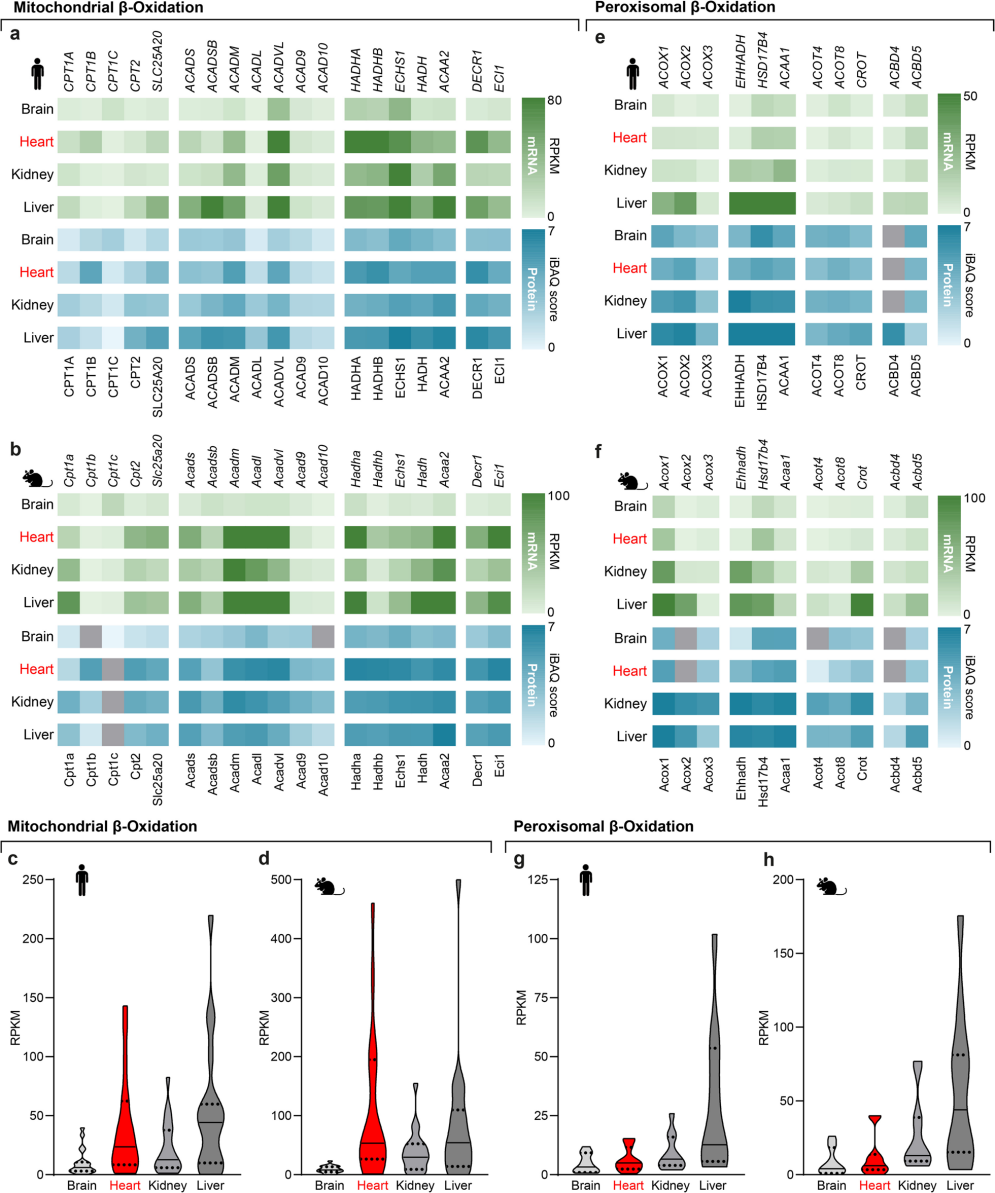
Expression of mitochondrial and peroxisomal β-oxidation factors. Mitochondrial β-oxidation factors are high in the liver and heart, whereas their peroxisomal counterparts are high in hepatic and renal tissue. **a–d** Transcriptome (green) and proteome (blue) data in **a** humans and **c** mice next to summarized distributions of transcriptome data in **b** human and **d** mice of factors involved in mitochondrial β-oxidation. **e–h** Heatmaps of the transcriptome (green) and proteome data (blue) in **e** humans and **g** mice with summarized distributions of transcriptome data in **f** human and **h** mice of peroxisomal β-oxidation factors

We compared tissue-specific gene expression data for *PXMP2*, *PXMP4*, *SLC25A17*, *PEX11,* and *PEX13*, and the ATP-binding cassette D (ABCD) family of FA transporters (Fig. 1a, b). Both sets are associated with metabolite flux. We found a uniformly distributed pattern (Figs. 2a, b and 3a–d), only *PXMP2* and *ABCD3* show increased expression in hepatic, but not cardiac tissue (Fig. 3a, b). ROS-protective CAT, EPHX2, and GSTKL1 correlate with mitochondrial β-oxidation factors and are lowest expressed in cerebral tissue (Figs. 3e–h and 4a-d). Cardiac *EPHX2* expression is low as well (Fig. 3e, f). D-aspartate oxidase (DDO) expression is highest in the heart, hinting at a potential tissue-specific function (Fig. 3e, f).

### Mitochondrial and peroxisomal β-oxidation

Mitochondrial and peroxisomal β-oxidation follow a similar sequence of reactions after FA activation: import, dehydrogenation, hydration, oxidation, thiolytic cleavage, and export. These steps are represented by organelle-specific substrates, enzymes, and products [35]. VLCFAs (C_22_-C_26_) are preferably metabolized by peroxisomes, mitochondria favor shorter medium- and long-chain FAs. Mitochondrial β-oxidation generates acetyl-CoA, FADH_2,_ and NADH for ATP production with intramitochondrial regeneration of FAD and NAD^+^. Peroxisomal β-oxidation produces acetyl-CoA, NADH, and hydrogen peroxide. Hydrogen peroxide is degraded by peroxisomal catalase (CAT), and NAD^+^ is regenerated by LDHBx- and MDH1x-dependent shuttles [52–54].

In our analysis, we selected organelle-specific enzymes catalyzing these steps. Additionally, we included the peroxisomal ER-tethering factors, ACBD4 and ACBD5*,* which are essential for ER-peroxisome contact sites and lipid transfer [55, 56] (Fig. 1b, d). Gene expression for mitochondrial β-oxidation is high in the heart and liver, but almost absent in the brain (Fig. 4a–d). HADHA and HADHB, together forming the Mitochondrial Trifunctional Protein (MTP), are markedly enriched in cardiac tissue (Fig. 4a, b). High MTP levels enhance mitochondrial ATP production in muscle tissue [57] by substrate tunneling [58], which emphasizes the cardiac dedication to ATP production. *ACBD5* gene expression is uniform across all tissues*,* and *ACBD4* is high in hepatic tissue (Fig. 4a, b). Peroxisomal β-oxidation genes are expressed highest in the liver, while cardiac, cerebral, and renal tissue show a comparably low expression (Fig. 4e–h).

### Peroxisomal α-oxidation

BCFAs contain methyl groups at C_β_ which sterically hinder β-oxidation. The most common BCFA is phytanic acid, a terpenoid-saturated FA derived from the chlorophyll side chain by gut microbiome fermentation of plant material in ruminant animals. Therefore, phytanic acid is abundant in dairy products and red meat [59]. The acyl-CoA synthetase SLC27A2 (also termed VLACS, FATP2) transports and activates BCFAs in peroxisomes and the ER, but not in mitochondria [60], highlighting the peroxisomal selectivity for BCFAs. Phytanoyl-CoA hydroxylase (PHYH) hydrates imported BCFA-CoAs at C_α_ [30], allowing cleavage of formyl-CoA by 2-hydroxyacyl-CoA lyase 1 (HACL1) to yield a fatty aldehyde accessible for β-oxidation. The following oxidation by the aldehyde dehydrogenase ALDH3A2 to an FA occurs exclusively in peroxisomes [61]. Peroxisomal α-methylacyl-CoA racemase (AMACR) converts naturally occurring (*R*)-stereoisomers of BCFAs or BC fatty aldehydes for subsequent (*S*)-selective β-oxidation [62].

**Fig. 5:**
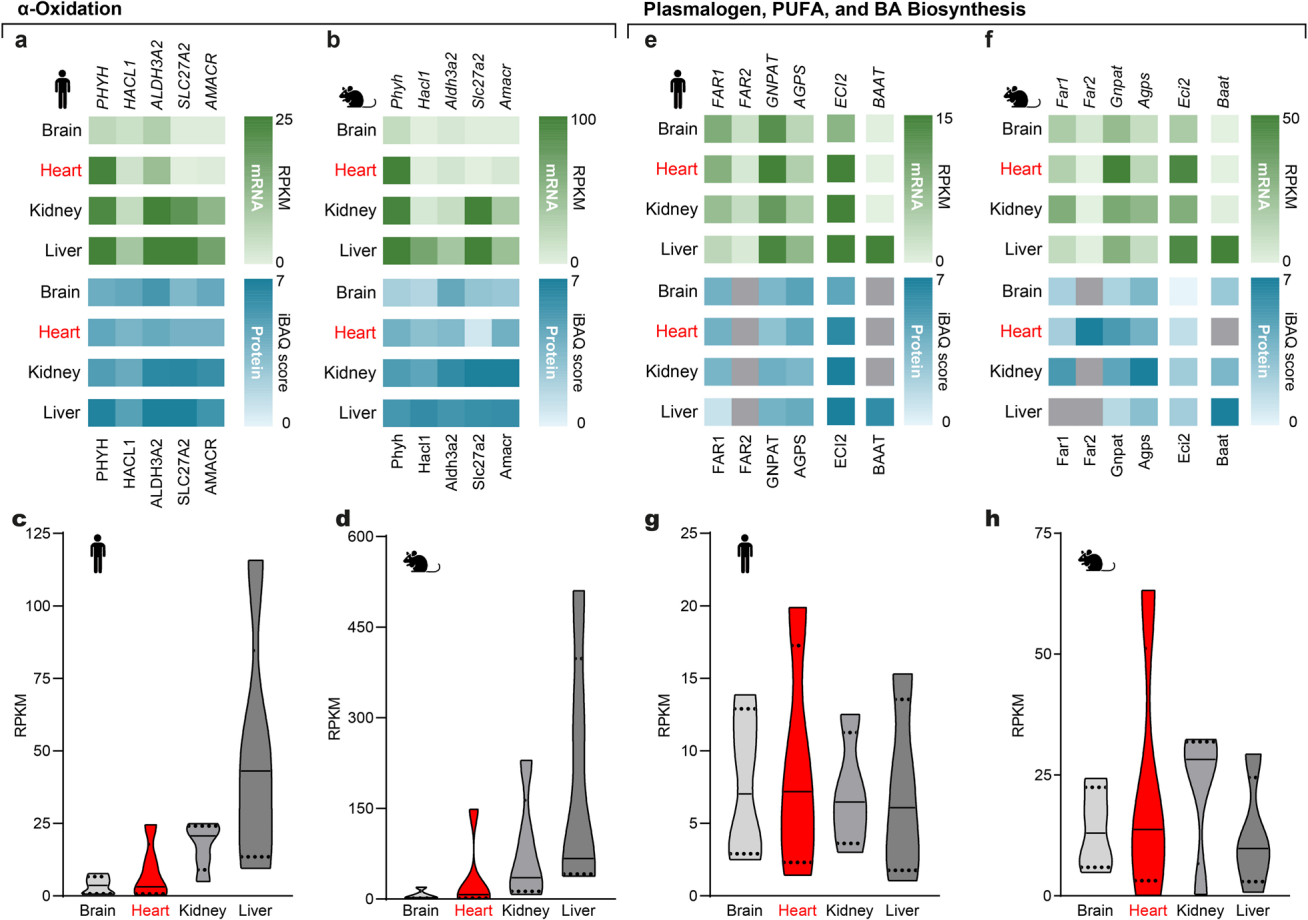
Expression profiles of α-oxidation and biosyntheses of plasmalogens, PUFAs, and BAs. α-Oxidation, BA, and PUFA biosynthesis factors are most abundant in the liver and least abundant in the brain, while factors for plasmalogen synthesis are highly expressed in cardiac tissue. **a–d** Transcriptome (green) and proteome data (blue) in **a** humans and **c** mice with summarized distributions of transcriptome data in **b** human and **d** mice of factors involved in peroxisomal α-oxidation. **e, g** Heatmaps showing transcriptomics (red) and proteomics (blue) in **e** humans and **g** mice of peroxisomal factors involved in plasmalogen, PUFA, and BA biosynthesis. **f, h** Aggregated distributions of transcriptome data in **f** human and **h** mice of peroxisomal factors involved in plasmalogen biosynthesis

We used the five peroxisomal factors to compare α-oxidation in different tissues (Fig. 1e). We included *ALDH3A2* despite differently localized isoforms due to its pathway specificity. Based on predominantly expressed α-oxidation enzymes in human and murine hepatic tissue (Fig. 5a–d), we confirmed the liver as a major site of BCFA degradation. Expression in the kidney is on a high level as well, whereas α-oxidation appears virtually absent in the brain (Fig. 5a–d). Interestingly, cardiac *PHYH* expression levels are high, without a comparable upregulation of the other gene products associated with α-oxidation, speaking for a cardiac-specific role of *PHYH* (Fig. 5a, b).

### Peroxisomal factors for biosynthesis of plasmalogens, PUFAs, and BAs

Peroxisomes are essential in the biosynthesis of plasmalogens, PUFAs, and BAs (Fig. 1f). Plasmalogens are glycerophospholipids with a vinyl ether group at the sn-1 position. This fatty alcohol is commonly saturated or mono-unsaturated. At the sn-2 position, plasmalogens typically contain PUFAs like docosahexaenoic acid (DHA) or arachidonic acid. Plasmalogens contribute to 20% of membrane phospholipids and make up more than half of the phosphatidylethanolamine in the brain, granulocytes, and the heart.

Plasmalogen biosynthesis initiates at peroxisomes and concludes at the ER. In peroxisomes, an activated acyl group is transferred to dihydroxyacetone phosphate (DHAP) by glycerone-phosphate O-acyltransferase (GNPAT) resulting in the ester-linked acyl-DHAP. Next, alkyl-dihydroxyacetone phosphate synthase (AGPS) exchanges the acyl-group of acyl-DHAP with a fatty alcohol producing the ether-linked alkyl-DHAP. Fatty alcohols are generated from acyl-CoA by peroxisomal FA reductases, FAR1 and FAR2. The subsequent steps for plasmalogen biosynthesis take place in the ER [21]. In our analysis, we selected the four factors catalyzing the initial reactions of plasmalogen biosynthesis in peroxisomes. We find the highest *GNPAT* expression in human and murine heart compared to the brain, liver, and kidney (Fig. 5e–h). Overall, the expression of peroxisomal factors for plasmalogen biosynthesis predominates in cardiac tissue (Fig. 5g, h), which reflects the heart-specific abundance of plasmalogens [23].

The biosynthesis of (V)LC-PUFAs, such as DHA, also involves the ER and peroxisomes. DHA is one of the major PUFAs in humans and is synthesized from dietary essential linolenic acid. Initially, cycles of linolenic acid desaturation and elongation produce tetracosahexaenoic acid (THA) in the ER, which then undergoes peroxisomal β-oxidation [19] to yield DHA. The β-oxidation of THA additionally involves the peroxisomal 3,2-trans-enoyl-CoA isomerase ECI2 [63]*.* Factors for peroxisomal β-oxidation are present in all tissues, and elevated in the liver (Fig. 4e–h). Both *ECI2* isoforms, having either a mitochondrial targeting signal and a PTS1, or a PTS1 only (*PECI*), are summarized in the collected source data. *ECI2* shows a basal expression in the brain and kidney, and an increase corresponding to high mitochondrial β-oxidation in cardiac and hepatic tissue (Fig. 5e, f), in line with a dual role in mitochondrial ATP production and peroxisomal PUFA biosynthesis.

BAs are synthesized from cholesterol in hepatocytes, transported through biliary ducts, and stored in the gall bladder, for intestinal secretion to support lipid digestion. Before transport, peroxisomal BA CoA-amino acid N-acyltransferase (BAAT) conjugates BAs with glycine or taurine to produce bile salts [20]. Accordingly, *BAAT* gene expression is exclusive in hepatic tissue (Fig. 5e, f).

### Peroxisomes in heart regions and cardiac cells

**Fig. 6:**
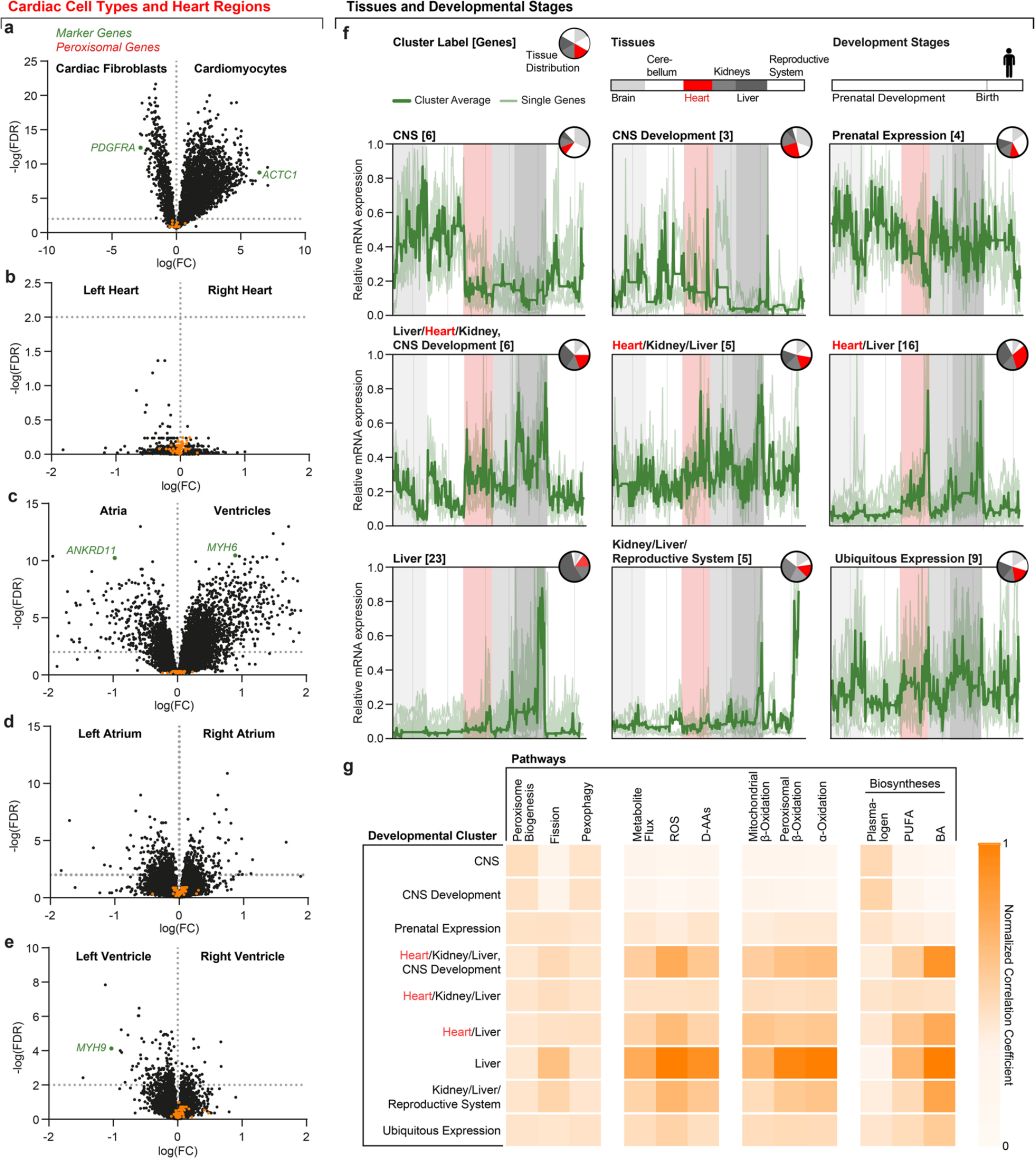
Differential gene expression in the heart and during tissue development. Human mitochondrial and peroxisomal genes show no differential expression in heart regions but during tissue development. **a–e** Logarithmic FDRs of logarithmic FCs from single-cell transcriptome data comparing **a** cardiac cell types, and **b–e** cardiomyocytes in different heart regions, with labeled reference (blue) and peroxisomal genes (red). Dotted gray horizontal lines indicate an FDR of 0.01. FDR, false-discovery rate; FC, fold change. **f** DTW-barycenter averages (dark green), and individual normalized mRNA expression (light green) in nine clusters across human development and tissue. The x-axis shows concatenated data for the analyzed tissues. The data is sorted by ascending developmental stages. Pie charts in the upper right corner indicate the tissue distribution per cluster. Bracketed numbers next to cluster labels display cluster size as the number of genes per cluster. **g** Normalized correlation coefficients of developmental clusters and pathways

Cardiac tissue composition varies between heart regions but includes mostly cardiomyocytes and fibroblasts [64]. A previous study reported a region-specific expression pattern of peroxisomal genes with a higher expression in the left ventricle by bulk tissue analysis [12]. As such, we analyzed our pathway-related gene sets using detailed single-cell mRNA expression data allocated to heart regions and cardiac cell types [39]. We calculated differential gene expression of full datasets between cardiomyocytes and fibroblasts, as well as left and right, atrial and ventricular regions. The comparison of cardiomyocytes and fibroblasts shows canonical, differentially expressed genes, e.g., the fibroblast-specific growth factor receptor *PDGFRA,* or *ACTC1* as a sarcomere component in cardiomyocytes (Fig. 6a) [64]. The gene expression patterns in region-specific cardiomyocytes are less well defined than between cardiac cell types (Fig. 6d). No significant differences were found when analyzing cardiomyocytes in left and right heart regions (Fig. 6b). Only when separating atrial and ventricular regions, differential gene expression in cardiomyocytes can be confirmed, e.g. by marker genes *ANKRD11*, *MYH6*, and *MYH9* (Fig. 6c–e) [64]. However, our peroxisomal gene set shows no enrichment in any comparison. Therefore, we cannot detect the previously reported differential expression of peroxisomal genes in heart regions on the single-cell level.

### Mitochondrial and peroxisomal gene expression during human tissue development

Our expression meta-analysis revealed a similar distribution of factors for peroxisome biogenesis proteins and metabolite flux across all tissues, and low or absent mitochondrial β-oxidation and α-oxidation expression in the brain. Peroxisomal β-oxidation and plasmalogen biosynthesis factors appear ubiquitously expressed but elevated in hepatic and cardiac tissue, respectively. We reasoned that metabolic tissue specifications of adult organisms may manifest during development by co-regulated and differential gene expression. Therefore, we analyzed mitochondrial and peroxisomal gene expression data across human tissues and developmental stages [37]. We added the available data from cerebellar and reproductive tissues to this analysis and used the set of 77 mitochondrial and peroxisomal genes (Additional file 1: Fig. S1). We clustered these data by unsupervised machine learning.

This analysis resulted in the organization of our target genes into nine developmental clusters, which we labeled based on tissue abundance and developmental trends (Fig. 6f; Additional file 2: Table S1): Nine genes, having either high or prenatal expression in the central nervous system (CNS, i.e. brain and cerebellum), aggregate in two groups (‘CNS’ and ‘CNS Development’). Four genes are prenatally expressed across most tissues (‘Prenatal Expression’). Two clusters include eleven genes with similar expression in the heart, kidney, liver, or during CNS development (‘Heart/Kidney/Liver, ‘CNS Development’ and ‘Heart/Kidney/Liver’). Most (39) genes show high expression in cardiac and hepatic tissue associated with two groups (‘Heart/Liver’ and ‘Liver’). The remaining 14 genes show either a high expression in hepatic, renal, and reproductive tissues or a ubiquitous expression (‘Kidney/Liver/Reproductive System’ and ‘Ubiquitous Expression’). Interestingly, *PEX7* is allocated to cardiac, and *PEX5* to highly expressed CNS genes (Table S1). Taken together, the developmental clusters show a differential gene expression in cardiac and hepatic tissue, ubiquitous genes, and co-regulated and CNS-specific developmental patterns (Fig. 6f; Additional file 2: Table S1).

Next, we correlated the tissue distribution of the nine developmental clusters and the twelve pathways of our expression meta-analysis (Fig. 6g). Peroxisome biogenesis, pexophagy, and plasmalogen biosynthesis show an even distribution across all developmental clusters. ‘CNS’ and ‘CNS development’ show only a low correlation with ROS, D-AAs, and FA metabolism. The genes allocated to ‘Prenatal Expression’, Heart/Kidney/Liver’, and ‘Ubiquitous Expression’ show similar correlations across all pathways. We find the strongest correlation between BA biosynthesis and ‘Liver’, indicating that BA synthesis is one of the most tissue-specific pathways under consideration. The developmental clusters with highly expressed genes in cardiac and hepatic tissue show a higher correlation with mitochondrial β-oxidation than clusters that include renal tissue. Peroxisomal FA oxidation pathways correlate with clusters, which show an abundance in renal, cardiac, and hepatic tissue (Fig. 6g). In summary, the cluster analysis indicates that developmental gene expression patterns regulate the tissue specificity of metabolic pathways in adults.

## Discussion

We uncovered distinct tissue-dependent expression patterns of mitochondrial and peroxisomal genes, revealing an interplay with specific anatomical and physiological contexts. Interestingly, these gene expression patterns emerged from a meta-analysis and developmental clustering across tissues but were not discernible in individual heart regions. This suggests a sophisticated regulatory network governing gene expression in a tissue-specific manner.

Factors involved in peroxisome biogenesis, fission, and pexophagy show comparable expression levels in all analyzed tissues and across developmental clusters, except for *PEX7*, which shows a preference for the heart. Cardiac tissue prioritizes PEX7-dependent import of PTS2-proteins, PHYH, ACAA1, and AGPS, essential enzymes for peroxisomal α- and β-oxidation, and plasmalogen synthesis, respectively.

We confirm the cerebral absence of FA oxidation and ROS-protective factors which are highly expressed in the heart, kidney, and liver [25]. FA oxidation provides acetyl-CoAs for ATP production and the synthesis of biomolecules. Mitochondrial β-oxidation enzymes show the highest expression in cardiac and hepatic tissue. Isotope studies indicate that peroxisomal acetyl-CoAs support ATP production in hepatocytes, but FA biosynthesis in cardiomyocytes [65, 66]. Across the analyzed tissues, peroxisomal FA oxidation factors are most expressed in the liver and kidney. Taken together, we find no considerable contribution of peroxisomal β-oxidation to cardiac energy metabolism.

Dietary FAs avoid hepatic first-pass metabolism and enter systemic circulation at the heart-proximal, left venous angle by lacteals, specific lymphatic vessels. This suggests that the dietary intake of peroxisomally metabolized BC/VLCFAs may prioritize the heart [67]. Single enzyme defects in peroxisomal α- and β-oxidation, including the PTS2-proteins, PHYH, and ACAA1, cause toxic levels of BC/VLCFAs leading to heart failure [14].

Mutations in AGPS*,* another *PEX7*-dependent PTS2-protein*,* also result in cardiomyopathies [68]. Together with the three other peroxisomal genes for plasmalogen biosynthesis, this pathway shows the highest expression in cardiac tissue. Plasmalogens contribute up to 32% of total glycerophospholipids in the heart and are significantly enriched within cardiac mitochondria [22, 23]. Emerging evidence suggests several functions for plasmalogens, e.g., phospholipid bilayer curvature [69], respirasome nanostructure, ATP production, and nucleotide metabolism [70], Ras-Raf-MAPK signaling [71], ferroptosis [72], hypoxia [73], and ROS [24], all of which are implicated in physiological heart function and cardiomyopathies [74]. Despite clear differential gene expression of reference genes, however, peroxisomal genes appear to show no preference for heart regions.

Collectively, we identified the relevance of plasmalogen synthesis in the heart and a cardiac priority for *PEX7* and PTS2-protein import, which are essential for peroxisomal α- and β-oxidation and correlate with cardiac manifestations of RCDP and RD. Experimental studies are required to ascertain whether peroxisomes are necessary for cardiac health and to assess the individual impact of metabolic pathways on normal and pathological heart function. These insights will further evolve the medical and clinical relevance of tissue-specific peroxisomal metabolism.

## Conclusion

Our findings reveal a similar capacity for peroxisome biogenesis across analyzed tissues, but differential expression patterns for mitochondrial and peroxisomal lipid metabolism. Cardiac peroxisomes are characterized by the most abundant expression of factors for plasmalogen biosynthesis, a high PEX7/PEX5 and PTS2/PTS1-protein ratio, and a basal level of peroxisomal α- and β-oxidation. We therefore suggest that peroxisomes are important for cardiac function and health. Research into these and related pathways and the underlying mechanisms may unveil novel therapeutic targets.

### Supplementary Information

**Additional file 1: Figure S1** Mitochondrial and peroxisomal gene expression across development and tissues.

**Additional file 2: Table S1** Peroxisomal proteomics, pathway-related genes, developmental clusters.

**Additional file 3: Table S2** Figure source data.

## Data Availability

All primary sources are publicly accessible [36–39]. We mapped each gene to unique database identifiers (ENSG, ENSMUSG, and UniProtKB AC/ID) (Additional file 2: Table S1). We included figure source data (Additional file 3: Table S2).

## Abbreviations

ABCD: ATP-binding cassette D
BA: Bile acid
BCFA: Branched-chain fatty acid
D-AA: D-amino acid
DHA: Docosahexaenoic acid
DHAP: Dihydroxyacetone phosphate
ER: Endoplasmic reticulum
FDR: False-discovery rate
HPA: Human Protein Atlas
iBAQ: Intensity-based absolute quantification
PBD: Peroxisome biogenesis disorder
*PEX*/PEX: Peroxin gene/protein
PTS1/2: Peroxisomal targeting signal 1/2
PUFA: Polyunsaturated fatty acid
RCDP: Rhizomelic chondrodysplasia punctata
RD: Refsum disease
RPKM: Reads per kilobase per million
FA: Fatty acid
ROS: Reactive oxygen species
VLCFA: Very-long-chain fatty acid
ZSS: Zellweger syndrome spectrum

## References

[1] Wanders RJA, Baes M, Ribeiro D, Ferdinandusse S, Waterham HR (2023). The physiological functions of human peroxisomes. Physiol Rev.

[2] Sargsyan Y, Thoms S (2020). Staying in healthy contact: How peroxisomes interact with other cell organelles. Trends Mol Med.

[3] Fujiki Y, Okumoto K, Honsho M, Abe Y (2022). Molecular insights into peroxisome homeostasis and peroxisome biogenesis disorders. Biochim Biophys Acta Mol Cell Res.

[4] Huber N, Guimaraes S, Schrader M, Suter U, Niemann A (2013). Charcot-Marie-Tooth disease-associated mutants of GDAP1 dissociate its roles in peroxisomal and mitochondrial fission. EMBO Rep.

[5] Eberhart T, Kovacs WJ (2018). Pexophagy in yeast and mammals: an update on mysteries. Histochem Cell Biol.

[6] Amberger JS, Bocchini CA, Scott AF, Hamosh A (2019). OMIM.org: leveraging knowledge across phenotype-gene relationships. Nucleic Acids Res..

[7] Karnati S, Baumgart-Vogt E (2008). Peroxisomes in mouse and human lung: their involvement in pulmonary lipid metabolism. Histochem Cell Biol.

[8] Morvay PL, Baes M, Van Veldhoven PP (2017). Differential activities of peroxisomes along the mouse intestinal epithelium. Cell Biochem Funct..

[9] Das Y, Swinkels D, Baes M (2021). Peroxisomal disorders and their mouse models point to essential roles of peroxisomes for retinal integrity. Int J Mol Sci.

[10] Muri J, Corak B, Matsushita M, Baes M, Kopf M (2022). Peroxisomes are critical for the development and maintenance of B1 and marginal zone B cells but dispensable for follicular B cells and T cells. J Immunol.

[11] Grings M, Tonin AM, Knebel LA, Zanatta Â, Moura AP, Filho CSD (2012). Phytanic acid disturbs mitochondrial homeostasis in heart of young rats: a possible pathomechanism of cardiomyopathy in Refsum disease. Mol Cell Biochem.

[12] Colasante C, Chen J, Ahlemeyer B, Baumgart-Vogt E (2015). Peroxisomes in cardiomyocytes and the peroxisome/peroxisome proliferator-activated receptor-loop. Thromb Haemost.

[13] Wanders RJA, Komen JC (2007). Peroxisomes, Refsum’s disease and the α- and ω-oxidation of phytanic acid. Biochem Soc Trans.

[14] Duker AL, Eldridge G, Braverman NE, Bober MB (2016). Congenital heart defects common in rhizomelic chondrodysplasia punctata. Am J Med Genet A.

[15] Wanders RJA (2013). Peroxisomes in human health and disease: metabolic pathways, metabolite transport, interplay with other organelles and signal transduction. Subcell Biochem.

[16] Waterham HR, Ferdinandusse S, Wanders RJA (2016). Human disorders of peroxisome metabolism and biogenesis. Biochim Biophys Acta.

[17] Zalckvar E, Schuldiner M (2022). Beyond rare disorders: a new era for peroxisomal pathophysiology. Mol Cell.

[18] Delille HK, Bonekamp NA, Schrader M (2006). Peroxisomes and disease: an overview. Int J Biomed Sci.

[19] Ferdinandusse S, Denis S, Mooijer PA, Zhang Z, Reddy JK, Spector AA (2001). Identification of the peroxisomal beta-oxidation enzymes involved in the biosynthesis of docosahexaenoic acid. J Lipid Res.

[20] Ferdinandusse S, Denis S, Faust PL, Wanders RJA (2009). Bile acids: the role of peroxisomes. J Lipid Res.

[21] Wanders RJ, Brites P (2010). Biosynthesis of ether-phospholipids including plasmalogens, peroxisomes and human disease: new insights into an old problem. Clin Lipidol.

[22] Dorninger F, Forss-Petter S, Wimmer I, Berger J (2020). Plasmalogens, platelet-activating factor and beyond: ether lipids in signaling and neurodegeneration. Neurobiol Dis.

[23] Gross RW (1985). Identification of plasmalogen as the major phospholipid constituent of cardiac sarcoplasmic reticulum. Biochemistry.

[24] Braverman NE, Moser AB (2012). Functions of plasmalogen lipids in health and disease. Biochim Biophys Acta (BBA) Mol Basis Dis.

[25] Schönfeld P, Reiser G (2013). Why does brain metabolism not favor burning of fatty acids to provide energy? Reflections on disadvantages of the use of free fatty acids as fuel for brain. J Cereb Blood Flow Metab.

[26] Lopaschuk GD, Ussher JR, Folmes CDL, Jaswal JS, Stanley WC (2010). Myocardial fatty acid metabolism in health and disease. Physiol Rev.

[27] Sack MN, Rader TA, Park S, Bastin J, McCune SA, Kelly DP (1996). Fatty acid oxidation enzyme gene expression is downregulated in the failing heart. Circulation.

[28] Violante S, Achetib N, van Roermund CWT, Hagen J, Dodatko T, Vaz FM (2019). Peroxisomes can oxidize medium- and long-chain fatty acids through a pathway involving ABCD3 and HSD17B4. FASEB J.

[29] Houten SM, Wanders RJA, Ranea-Robles P (2020). Metabolic interactions between peroxisomes and mitochondria with a special focus on acylcarnitine metabolism. Biochim Biophys Acta (BBA) Mol Basis Dis.

[30] Jansen GA, Wanders RJA (2006). Alpha-oxidation. Biochim Biophys Acta (BBA) Mol Cell Res.

[31] Yifrach E, Fischer S, Oeljeklaus S, Schuldiner M, Zalckvar E, Warscheid B (2018). Defining the mammalian peroxisomal proteome. Subcell Biochem.

[32] Binder JX, Pletscher-Frankild S, Tsafou K, Stolte C, O’Donoghue SI, Schneider R (2014). COMPARTMENTS: unification and visualization of protein subcellular localization evidence. Database.

[33] UniProt Consortium (2023). UniProt: the universal protein knowledgebase in 2023. Nucleic Acids Res.

[34] Wanders RJA, Waterham HR (2006). Biochemistry of mammalian peroxisomes revisited. Annu Rev Biochem.

[35] Houten SM, Violante S, Ventura FV, Wanders RJA (2016). The biochemistry and physiology of mitochondrial fatty acid β-oxidation and its genetic disorders. Annu Rev Physiol.

[36] Karlsson M, Zhang C, Méar L, Zhong W, Digre A, Katona B (2021). A single-cell type transcriptomics map of human tissues. Sci Adv..

[37] Cardoso-Moreira M, Halbert J, Valloton D, Velten B, Chen C, Shao Y (2019). Gene expression across mammalian organ development. Nature.

[38] Samaras P, Schmidt T, Frejno M, Gessulat S, Reinecke M, Jarzab A (2020). ProteomicsDB: a multi-omics and multi-organism resource for life science research. Nucleic Acids Res.

[39] Kanemaru K, Cranley J, Muraro D, Miranda AMA, Ho SY, Wilbrey-Clark A (2023). Spatially resolved multiomics of human cardiac niches. Nature.

[40] Grubbs FE (1950). Sample criteria for testing outlying observations. Ann Math Stat.

[41] Holm S (1979). A simple sequentially rejective multiple test procedure. Scand J Stat.

[42] Benjamini Y (2010). Discovering the false discovery rate. J R Stat Soc Ser B (Stat Methodol).

[43] Kunze M (2020). The type-2 peroxisomal targeting signal. Biochim Biophys Acta (BBA) Mol Cell Res.

[44] Okumoto K, Kametani Y, Fujiki Y (2011). Two proteases, trypsin domain-containing 1 (Tysnd1) and peroxisomal lon protease (PsLon), cooperatively regulate fatty acid β-oxidation in peroxisomal matrix. J Biol Chem.

[45] Mizuno Y, Ninomiya Y, Nakachi Y, Iseki M, Iwasa H, Akita M (2013). Tysnd1 deficiency in mice interferes with the peroxisomal localization of PTS2 enzymes, causing lipid metabolic abnormalities and male infertility. PLoS Genet.

[46] Thoms S, Erdmann R (2005). Dynamin-related proteins and Pex11 proteins in peroxisome division and proliferation. FEBS J.

[47] Thoms S, Gärtner J (2012). First PEX11β patient extends spectrum of peroxisomal biogenesis disorder phenotypes. J Med Genet.

[48] Woodyatt NJ, Lambe KG, Myers KA, Tugwood JD, Roberts RA (1999). The peroxisome proliferator (PP) response element upstream of the human acyl CoA oxidase gene is inactive among a sample human population: significance for species differences in response to PPs. Carcinogenesis.

[49] Lawrence JW, Li Y, Chen S, DeLuca JG, Berger JP, Umbenhauer DR (2001). Differential gene regulation in human versus rodent hepatocytes by peroxisome proliferator-activated receptor (PPAR) alpha. PPAR alpha fails to induce peroxisome proliferation-associated genes in human cells independently of the level of receptor expresson. J Biol Chem.

[50] James C, Lenz C, Urlaub H, Kehlenbach RH (2021). Sequestosome 1 is part of the interaction network of VAPB. Int J Mol Sci.

[51] Wang W, Xia Z-J, Farré J-C, Subramani S (2017). TRIM37, a novel E3 ligase for PEX5-mediated peroxisomal matrix protein import. J Cell Biol.

[52] Chornyi S, Costa CF, Ijlst L, Fransen M, Wanders RJA, van Roermund CWT (2023). Human peroxisomal NAD+/NADH homeostasis is regulated by two independent NAD(H) shuttle systems. Free Radic Biol Med.

[53] Schueren F, Lingner T, George R, Hofhuis J, Dickel C, Gärtner J (2014). Peroxisomal lactate dehydrogenase is generated by translational readthrough in mammals. Elife.

[54] Baumgart E, Fahimi HD, Stich A, Völkl A (1996). L-lactate dehydrogenase A4- and A3B isoforms are bona fide peroxisomal enzymes in rat liver. Evidence for involvement in intraperoxisomal NADH reoxidation. J Biol Chem.

[55] Costello JL, Castro IG, Schrader TA, Islinger M, Schrader M (2017). Peroxisomal ACBD4 interacts with VAPB and promotes ER-peroxisome associations. Cell Cycle.

[56] Hua R, Cheng D, Coyaud É, Freeman S, Di Pietro E, Wang Y (2017). VAPs and ACBD5 tether peroxisomes to the ER for peroxisome maintenance and lipid homeostasis. J Cell Biol.

[57] Kappler L, Hoene M, Hu C, von Toerne C, Li J, Bleher D (2019). Linking bioenergetic function of mitochondria to tissue-specific molecular fingerprints. Am J Physiol Endocrinol Metab.

[58] Wang Y, Mohsen A-W, Mihalik SJ, Goetzman ES, Vockley J (2010). Evidence for physical association of mitochondrial fatty acid oxidation and oxidative phosphorylation complexes. J Biol Chem.

[59] Hellgren LI (2010). Phytanic acid–an overlooked bioactive fatty acid in dairy fat?. Ann N Y Acad Sci.

[60] Wanders RJA, Waterham HR, Ferdinandusse S (2015). Metabolic Interplay between peroxisomes and other subcellular organelles including mitochondria and the endoplasmic reticulum. Front Cell Dev Biol.

[61] Jansen GA, van den Brink DM, Ofman R, Draghici O, Dacremont G, Wanders RJ (2001). Identification of pristanal dehydrogenase activity in peroxisomes: conclusive evidence that the complete phytanic acid alpha-oxidation pathway is localized in peroxisomes. Biochem Biophys Res Commun.

[62] Kong G, Lee H, Tran Q, Kim C, Gong N, Park J (2020). Current knowledge on the function of α-methyl acyl-CoA racemase in human diseases. Front Mol Biosci.

[63] Geisbrecht BV, Zhang D, Schulz H, Gould SJ (1999). Characterization of PECI, a novel monofunctional Delta(3), Delta(2)-enoyl-CoA isomerase of mammalian peroxisomes. J Biol Chem.

[64] Litviňuková M, Talavera-López C, Maatz H, Reichart D, Worth CL, Lindberg EL (2020). Cells of the adult human heart. Nature.

[65] Reszko AE, Kasumov T, David F, Jobbins KA, Thomas KR, Hoppel CL (2004). Peroxisomal fatty acid oxidation is a substantial source of the acetyl moiety of malonyl-CoA in rat heart. J Biol Chem.

[66] Bian F, Kasumov T, Thomas KR, Jobbins KA, David F, Minkler PE (2005). Peroxisomal and mitochondrial oxidation of fatty acids in the heart, assessed from the 13C labeling of malonyl-CoA and the acetyl moiety of citrate. J Biol Chem.

[67] Ding L, Sun W, Balaz M, He A, Klug M, Wieland S (2021). Peroxisomal β-oxidation acts as a sensor for intracellular fatty acids and regulates lipolysis. Nat Metab.

[68] David C, Ouimet B, Goulet C, De Loof M, Alikashani A, Daneault C (2021). A targeted knockdown of Agps in H9c2 cells lowered the level of plasmalogen lipids that disturbed mitochondrial function. Arch Cardiovasc Dis Suppl.

[69] Koivuniemi A (2017). The biophysical properties of plasmalogens originating from their unique molecular architecture. FEBS Lett.

[70] Bennett CF, O’Malley KE, Perry EA, Balsa E, Latorre-Muro P, Riley CL (2021). Peroxisomal-derived ether phospholipids link nucleotides to respirasome assembly. Nat Chem Biol.

[71] Huang F, Cai F, Dahabieh MS, Gunawardena K, Talebi A, Dehairs J (2023). Peroxisome disruption alters lipid metabolism and potentiates antitumor response with MAPK-targeted therapy in melanoma. J Clin Invest.

[72] Zou Y, Henry WS, Ricq EL, Graham ET, Phadnis VV, Maretich P (2020). The plasticity of ether lipids promotes ferroptosis susceptibility and evasion. Nature.

[73] Jain IH, Calvo SE, Markhard AL, Skinner OS, To T-L, Ast T (2020). Genetic screen for cell fitness in high or low oxygen highlights mitochondrial and lipid metabolism. Cell.

[74] Fang X, Wang H, Han D, Xie E, Yang X, Wei J (2019). Ferroptosis as a target for protection against cardiomyopathy. Proc Natl Acad Sci USA.

