# Supplementary figures and images for: Tissue-specific roles of peroxisomes revealed by expression meta-analysis
Source: Biol Direct. 2024 Feb 16;19:14. doi: 10.1186/s13062-024-00458-1 (PMC10873952; doi:10.1186/s13062-024-00458-1)

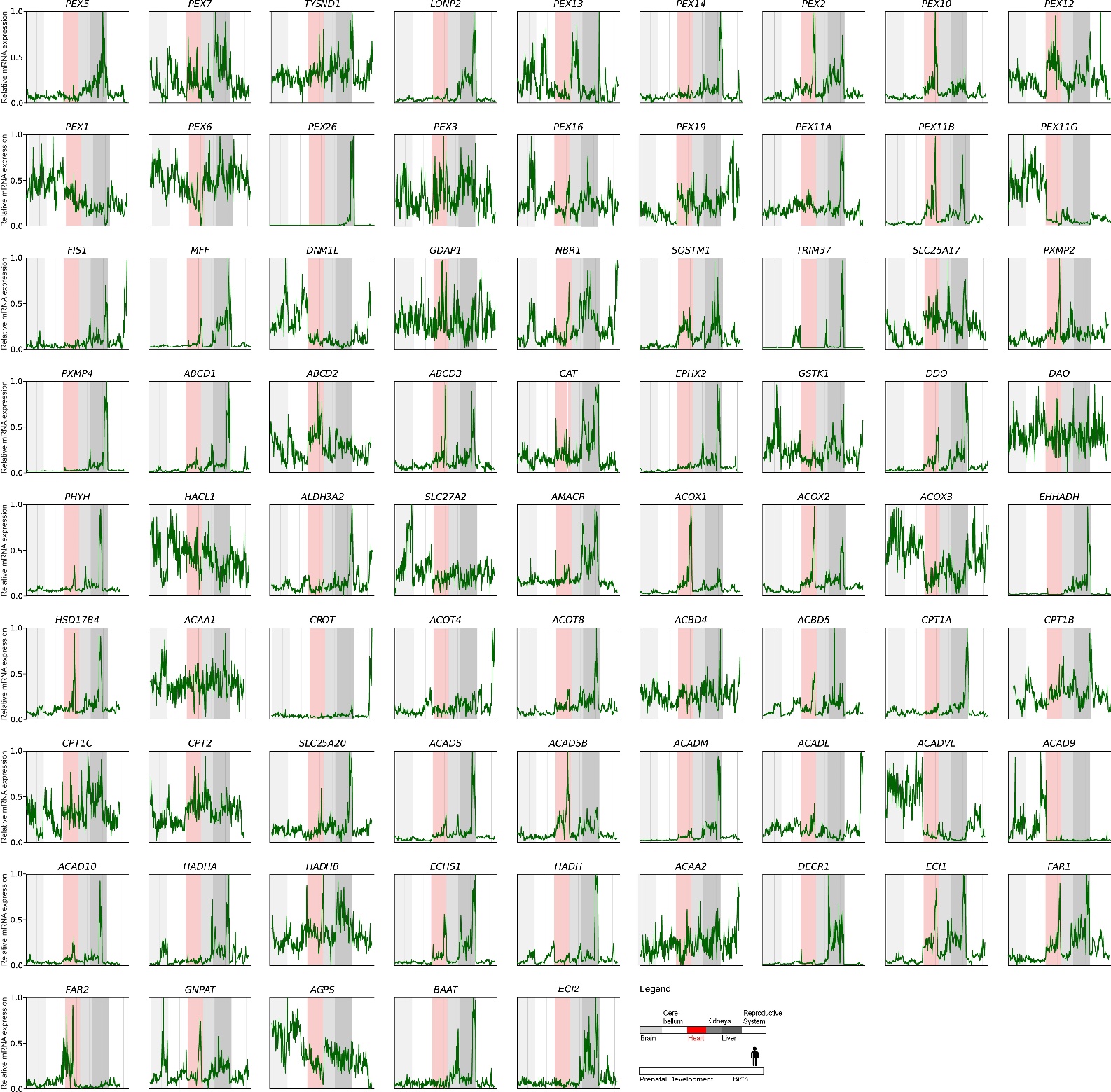


Figure S1

Supplement: Supplementary file 1 — Additional file 1: Figure S1 Mitochondrial and peroxisomal gene expression across development and tissues. [file 13062_2024_458_MOESM1_ESM.docx]
